# IMPORTANT COMMUNITY FEATURES AND SERVICES IN DIVERSE OLDER ADULTS

**DOI:** 10.1093/geroni/igad104.2362

**Published:** 2023-12-21

**Authors:** Kyeongmo Kim, Andy Hong

**Affiliations:** Virginia Commonwealth University, Richmond, Virginia, United States; University of Utah, Salt Lake City, Utah, United States

## Abstract

Many cities and towns have tried to create neighborhood environments favorable to older adults. However, it raises the question of whether community aging initiatives reflect the diverse needs of older adults. This study examined how much different types of neighborhood physical and social environments were essential to the life of diverse older adults. This study used data from the 2018 AARP Home and Community Preferences Survey with 762 adults aged 50 and older in the USA (female: 50%; White: 70%, Black: 17%, Hispanic: 12%, other race: 5%). The outcome variables were participants’ rates on the relative importance of seven community features (i.e., outdoor space, housing, transportation, street, social participation, civic engagement, and job). We ran multiple regression analyses to examine the relationships between the importance rating of community features and diverse older populations. Women thought all community features were important in their daily life compared to men, except for transportation. Black and Hispanic older adults valued all seven community features more highly than White older adults. People with disabilities thought housing and civic engagement were essential daily; people who experienced loneliness thought housing, transportation, social participation, and civic engagement were important. This study adds to the growing literature on identifying the diverse needs of older adults in developing and creating age-friendly environments. Community features and services were essential to women and racial and ethnic minority adults. Depending on older adults’ demographic and health-related characteristics, policymakers and practitioners should develop a plan to reflect the diverse needs of older adults.

